# A concise classification of *bencao* (*materia medica*)

**DOI:** 10.1186/s13020-018-0176-y

**Published:** 2018-04-10

**Authors:** Zhongzhen Zhao, Ping Guo, Eric Brand

**Affiliations:** School of Chinese Medicine, Hong Kong Baptist University, Kowloon Tong, Hong Kong China

**Keywords:** *Bencao* (*materia medica*), Chinese medicinal authentication, Traditional Chinese medicine

## Abstract

Books that record the sources and applications of medicinal materials are commonly known as *bencao* (*materia medica*) in China. *Bencao* (*materia medica*) literature review is the very first step in the standard authentication procedure of Chinese medicinals. As an important part of China’s cultural heritage, these various *bencao* (*materia medica*) texts represent centuries of accumulated wisdom in combating disease and preserving health. In this short review, *bencao* (*materia medica*) classics of China are broadly divided into three major categories in our routine practice: mainstream *bencao* (*materia medica*), thematic *bencao* (*materia medica*) and regional *bencao* (*materia medica*). The overall significance and current situation of exploration of *bencao* (*materia medica*) literature are summarized as well.

## Background

**Fig. 1:**
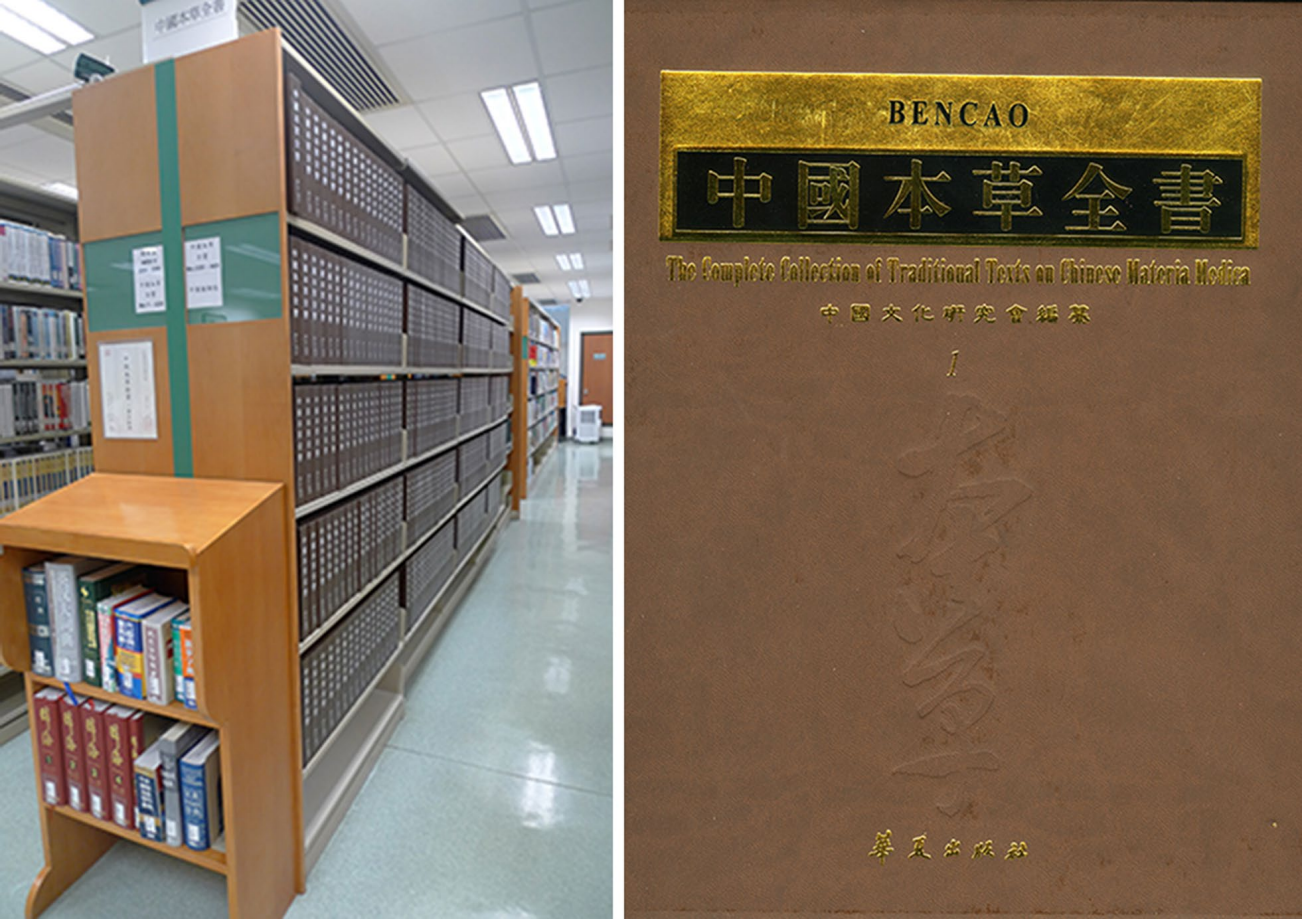
*The Complete Collection of Traditional Texts on Chinese Materia Medica* at the Hong Kong Baptist University Library

China is a large country with diverse ecological conditions and abundant botanical, zoological and mineral resources. Among them, some are of medicinal value and have been used medicinally since ancient times. In China, books that record the sources and applications of medicinal materials are commonly known as *bencao* (*materia medica*). The Chinese term “*bencao*”, which literally means “rooted in herbs”, reflects the fact that most medicinal materials are derived from botanical sources. *Bencao* (*materia medica*) texts of past dynasties primarily describe three aspects of Chinese medicinals: medicinal materials, medicinal properties, and medicinal principles. As an important part of China’s cultural heritage, these various *bencao* (*materia medica*) texts represent centuries of accumulated wisdom in combating disease and preserving health. *The Complete Collection of Traditional Texts on Chinese Materia Medica*, a 410-volume and 246,000-page collection complied by the Association of Chinese Culture Research, includes more than 800 *bencao* (*materia medica*) classics from 220 BC to 1911 AD. This collection highlights the value of traditional Chinese medicine (TCM) as a rich source for knowledge-based medical rediscovery due to its documentation of clinical experiences over thousands of years, and also illustrates the monumental challenge of selecting the best parts of TCM for modern innovation ([1–3], Fig. 1).

**Fig. 2:**
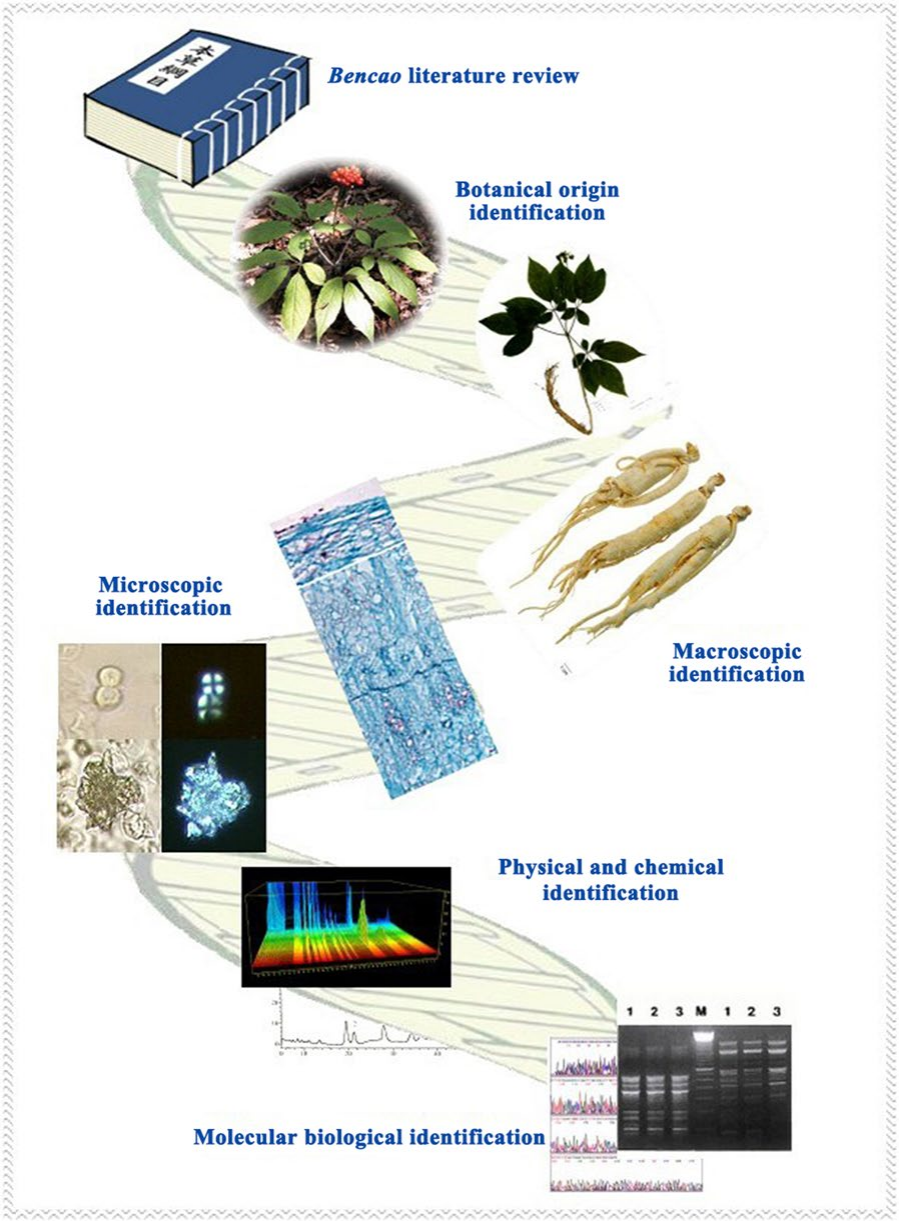
Standard authentication procedure for Chinese medicinals

The genre of *bencao* (*materia medica*) literature is uniquely developed in Chinese medicine, and represents a tremendous historical and cultural resource as well as an important reference point for clinicians, medical historians, and scientists in disciplines such as new drug discovery and Chinese medicinal authentication. Authentication is fundamental for Chinese medicinal standardization, and *bencao* (*materia medica*) literature review is the very first step in the standard authentication procedure of Chinese medicinals ([4], Fig. 2).

In practice, *bencao* (*materia medica*) classics of China are broadly divided into three major categories: (a) mainstream *bencao* (*materia medica*): the most influential *bencao* (*materia medica*) classics from key historical periods, (b) thematic *bencao* (*materia medica*): specialized *bencao* (*materia medica*) texts dedicated to specific topics, and c) regional *bencao* (*materia medica*): *bencao* (*materia medica*) texts focused on medicinal materials from specific regions.

## Mainstream *bencao* (*materia medica*)

Over the past 2000 years, five monumental works stand out in the genre of *bencao* (*materia medica*) literature. They are the most influential classics from key historical periods.

1. *The Divine Husbandman’s Classic of Materia Medica* (*Shen Nong Ben Cao Jing*) This is the earliest extant *bencao* (*materia medica*) text, compiled in the Eastern Han Dynasty (25–220 AD). This text records 365 medicinals and summarizes medicinal experiences up to the Han Dynasty. Medicinals are classified into three categories (high-grade, medium-grade and low-grade) based on their medicinal effects and toxicity. Entries for each medicinal substance include nomenclature, properties, compatibilities, and medical applications. Descriptions of production regions and the ecological environment of some medicinal plants are also recorded briefly.
2. *Collection of Commentaries on the Classic of the Materia Medica* (*Ben Cao Jing Ji Zhu*) Tao Hongjing, a physician of the North and South Kingdoms period (420–589 AD), compiled this text by preserving and annotating *The Divine Husbandman’s Classic of Materia Medica* (*Shen Nong Ben Cao Jing*) and adding another 365 medicinals. It records 730 medicinals and established the framework of *bencao* (*materia medica*) compilations adopted by later generations. In this book, medicinal substances are further classified into seven categories based on their natural properties: jades/stones, herbs, trees, insects/beasts, fruits/vegetables, crops, and medicinals with names but without actual applications.
3. *Newly Revised Materia Medica* (*Xin Xiu Ben Cao*) In 659 AD, commissioned by the government of the Tang Dynasty (618–907 AD), this text records 850 medicinals and is considered to be the earliest national pharmacopoeia in China.
4. *Materia Medica Arranged According to Pattern* (*Zheng Lei Ben Cao*) Compiled by a physician named Tang Shenwei and published in 1108 AD, this is the most praiseworthy *bencao* (*materia medica*) of the Song Dynasty (960–1279 AD) as it comprehensively summarizes herbal knowledge up to that time. There are three versions of this book (“*Da Guan*”, “*Zheng He*” and “*Shao Xing*”) currently in circulation. It records 1746 medicinals and is the only *bencao* (*materia medica*) text from the Song and previous dynastic periods that survived intact. It features clearly preserved quotations from previous works and stands out as an important reference point for *bencao* (*materia medica*) knowledge leading up to the Song Dynasty.
5. *Compendium of Materia Medica* (*Ben Cao Gang Mu*) Written by Li Shizhen, a physician of the Ming Dynasty (1368–1644 AD), this text was first published in 1596. It records 1892 medicinal substances. This massive and influential compilation represents the highest academic achievement among all the ancient Chinese *bencao* (*materia medica*). It not only comprehensively summarized medical knowledge up to the sixteenth century in China, but also contributed greatly to the development of natural sciences in the world.

## Thematic *bencao* (*materia medica*)

Thematic *bencao* (*materia medica*) refer to specialized *bencao* (*materia medica*) texts dedicated to specific topics, such as medicinal processing, authentication, dietary therapy, and medicinal properties. Examples include *Grandfather Lei*’*s Treatise on Herbal Processing* (*Lei Gong Pao Zhi Lun*) and *Origins of the Materia Medica* (*Ben Cao Yuan Shi*). The former is the first monograph on Chinese medicinal processing, written in about 500 AD in the North and South Kingdoms period; it summarizes the literature and experiences of the ancient practice of processing. The latter stands out in the history of *bencao* (*materia medica*) as an outstanding monograph on macroscopic identification, written by Li Zhongli in the Ming Dynasty (1368–1644 AD). It is characterized by detailed illustrations and descriptions of diagnostic features of raw medicinal materials. Other examples of thematic *bencao* texts include the *Materia Medica of Dietary Therapy* (*Shi Liao Ben Cao*) of the Tang Dynasty and the *Materia Medica for Decoctions* (*Tang Ye Ben Cao*) of the Yuan Dynasty (1279–1368 AD). The former was dedicated to the theory and practice of dietary therapy, and the latter to the theory of medicinal properties and clinical experience.

## Regional *bencao* (*materia medica*)

Regional *bencao* (*materia medica*) texts record knowledge related to medicinal substances derived from specific local regions. Examples include the *Materia Medica from Steep Mountainsides* (*Lu Chan Yan Ben Cao*) and the *Essentials of Raw Herbs in Lingnan* (*Sheng Cao Yao Xing Bei Yao*). The former was the first regional *bencao* (*materia medica*) with color illustrations dedicated to local medicinal plants; compiled in 1220 AD in the Song Dynasty, it focused on the area around modern-day Hangzhou. The latter was compiled in 1711 AD in the Qing Dynasty (1644–1911 AD); it records botanical medicinals used in the Lingnan region, a geographic area located in the southern part of China.

## Conclusion

*Bencao* (*materia medica*) tradition provides a rich record of knowledge that has been gradually refined for centuries, opening a window into the cultural tradition of scholarship and textual research that defines TCM. *Bencao* (*materia medica*) literature such as the *Compendium of Materia Medica* (*Ben Cao Gang Mu*) illustrates important developments in the broader history of natural sciences in China, and this text has been translated into European languages since 1735 AD. In recent decades, exploration of *bencao* (*materia medica*) literature has facilitated dramatic medical discoveries, such as the anti-malarial drug artemisinin from sweet wormwood (*qinghao*, Artemisiae Annuae Herba) [5].

However, at present, the overall significance of *bencao* (*materia medica*) literature remains underestimated outside of the TCM community. Therefore, in addition to other aspects of TCM, attention is needed to preserve the cultural resources that lie at the heart of *bencao* (*materia medica*).

Although our research efforts related to *daodi* medicinal materials, medicinal authentication and Chinese medicinal processing are closely connected to *bencao* (*materia medica*) literature [6–9], the future of *bencao* (*materia medica*) research depends upon a multidisciplinary approach that protects the past while embracing the future.

## Abbreviation

TCM: traditional Chinese medicine
